# The effects of synbiotic and/or vitamin D supplementation on gut-muscle axis in overweight and obese women: a study protocol for a double-blind, randomized, placebo-controlled trial

**DOI:** 10.1186/s13063-022-06598-x

**Published:** 2022-08-04

**Authors:** Sanaz Jamshidi, Seyed Jalil Masoumi, Behnaz Abiri, Mohammadreza Vafa

**Affiliations:** 1 Department of Nutrition, School of Public Health, Iran University of Medical Sciences, Hemat expressway, P.O.BOX: 1449614535, Tehran, Iran; 2 Nutrition Research Center, Department of Clinical Nutrition, School of Nutrition and Food Sciences, Shiraz University of Medical Sciences, Shiraz, Iran; 3 Obesity Research Center, Research Institute for Endocrine Sciences, Shahid Beheshti University of Medical Sciences, Tehran, Iran

**Keywords:** Synbiotic, Vitamin D, Microbiome, Sarcopenia, Muscle mass, Obese women

## Abstract

**Background:**

Sarcopenia refers to an age-related loss of skeletal muscle content, strength, and function, leading to a decrease in mobility. Obesity may exacerbate age-related complications such as sarcopenia through inflammatory pathways. In addition, intestinal dysbiosis has been proposed as an emerging contributor to sarcopenia due to the stimulation of the immune system and elevated barrier permeability of the intestine. Targeting microbiome with synbiotic and vitamin D supplementation may modulate the microbiome followed by the enhancement of sarcopenia indices. Thus, the present study aims to evaluate the effect of synbiotic supplementation with or without vitamin D on the intestinal microbiome and its relationship with strength, muscle function, and body composition in middle-aged overweight and obese women.

**Methods:**

This multi-factorial, double-blind, randomized controlled trial will be conducted on 88 participants in eight weeks. The participants will be allocated into four groups receiving vitamin D placebo (weekly) and synbiotic placebo (daily), vitamin D and synbiotic placebo, vitamin D placebo and symbiotic, and vitamin D and synbiotic. Intestinal microbiome assessment will be done by DNA isolation and real-time polymerase chain reaction (PCR). In addition, anthropometric indices, body composition, muscle strength, and physical performance will be evaluated by standard methods. All measurements will be made at the beginning and end of the study.

**Discussion:**

The previous studies showed that probiotics were involved in reducing inflammation, insulin sensitivity, modulation of atrophy markers such as atherogen-1, and decreasing reactive oxygen indices. In addition, vitamin D was found to improve the intestinal microbiome and facilitate muscle anabolism. The present protocol is novel as it aims to investigate the impact of the co-supplementation of synbiotic and vitamin D on the gut microbiome and sarcopenia indices.

**Trial registration:**

This trial has been registered in the Iranian Registry of Clinical Trials (IRCT20090822002365N25, date of registration: March 2021).

## Background

Sarcopenia has been described as the involuntary loss of skeletal muscle mass content and impairment of muscle function, which is accompanied by mobility decline, physical disability, decreased standing balance, high rate of hospitalization, and mortality [1, 2]. Sarcopenia, as a widespread condition, is prevalent in almost 15% of healthy elderly population up to 76% of hospitalized older adults [3]. Recent evidence has revealed the prevalence of sarcopenia to be approximately 21% among Iranian older adults [4]. This syndrome is accompanied by a wide burden and is rapidly influenced by increased age [5]. On the other hand, obesity may affect age-related complications, leading to sarcopenia by activating inflammatory pathways [6].

The pathogenesis of sarcopenia involves various aspects including replacement of fat instead of muscle fibers, muscle metabolism changes, neuromuscular junction degeneration, fibrosis increments, and oxidative stress [7]. Moreover, intestinal microbiome is an emerging contributor to health and diseases, and its interaction with different organs such as brain and liver has been investigated extensively. The possible communication between the gut microbiota and muscle has been posited recently as a “gut-muscle axis” [8]. Healthy gut microbiota can modulate physiological processes including the downregulation of inflammatory biomarkers and inflamm-aging, antioxidant capacity enhancement, insulin sensitivity improvement by the production of diet-derived polyphenols, B complex vitamins (B2, B9 and B12), ellagitannins, and short-chain fatty acids (SCFA), increased resistance to fatigue, enhanced mitochondrial biogenesis and energy production, stimulation of anabolism and cell production, gut barrier maintenance, and reduction of xenobiotic absorption [9–11].

Disruptions in the gut microbiome account for the pathogenesis of many complications. Furthermore, evidence has demonstrated that sarcopenia and physical weakness have been associated with intestinal dysbiosis due to the stimulation of the immune system, elevated barrier permeability of the intestine, and increased lipopolysaccharides levels [12]. Ultimately, this condition leads to muscle loss by creating a systemic inflammatory state. In addition, it has been shown in animal models that intestinal dysbiosis can disrupt neuromuscular transmission, leading to muscle protein catabolism [13]. Moreover, it should be noted that aging and obesity have similar consequences, and their synergistic influence on muscle function may exacerbate the health status [14]. Aging is correlated to gut microbiota alterations, and this shift is accompanied by bacterial diversity decline from Firmicutes, Bifidobacterium, and Bacteroides reduction to the expansion of opportunistic bacteria that have the potential to induce systemic inflammation [15]. On the other hand, diets with high sugar and fat contents can affect the pathways related to histidine, glutamate, SCFAs, macrophage activation, nuclear factor kappa B (NF-κB), and toll-like receptor (TLR) and change the intestinal microbiome balance, which is common in obesity, metabolic syndrome, and diabetes [16].

Targeting gut microbiota with nutritional supplements is a favorable approach for modulating the microbiome followed by the enhancement of muscle mass and function. For instance, dietary interventions with probiotics, prebiotics, and synbiotics facilitate weight loss/maintenance in obese individuals through microbiome improvements [17]. Probiotics also contribute to muscle physiology via increased access to amino acids, suppression of inflammatory cytokine production, and effects on bile acid metabolites [18]. In addition to synbiotics, vitamin D improves the intestinal microbiome function and homeostasis by downregulating inflammation, innate immunity enhancement, and intestinal barrier maintenance [19]. Some other mechanisms proposed for vitamin D include increasing the production of antimicrobial peptides (beta-defensin and cathelicidin) by macrophages, regulating the expression of tight junctions and adhesive binding proteins, and suppressing epithelial cell apoptosis that generally maintains the mucosal barrier function [20]. Vitamin D receptors (VDR) also play an important role in protecting probiotics against inflammation [21]. On the other hand, probiotics can have a synergistic effect with vitamin D by improving the expression of VDR [22, 23]. Overall, dietary interventions with synbiotic and vitamin D may play an essential role in modulating the intestinal microbiome. Following that, the healthy microbiome can be a mediator between nutrition and phenotypes associated with aging and obesity.

Based on what was mentioned above, it is vitally important to consider all the possible factors affecting the initiation of sarcopenia and investigate efficient interventions in line with these pathogeneses. Since synbiotics and vitamin D play different roles in improving the microbiome and no study has been done on the effects of these supplements on the gut-muscle axis, gut microbiome, and sarcopenia indices, the present study aims to evaluate the effect of synbiotic supplementation with or without vitamin D on the intestinal microbiome and their relationships with strength, muscle function, and body composition in middle-aged overweight and obese women.

## Methods

### Design

**Fig. 1:**
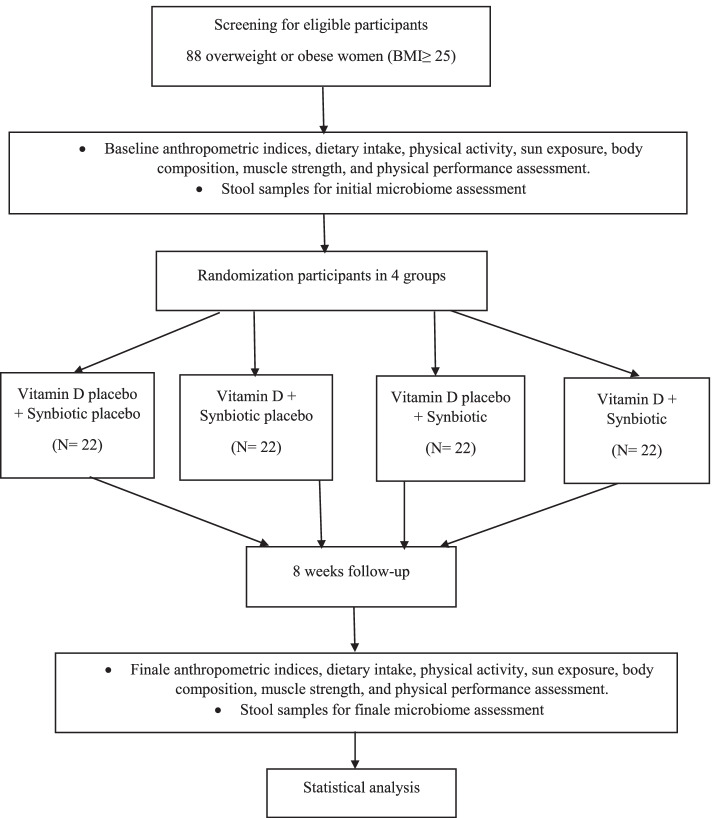
Flow diagram of the study protocol

**Fig. 2:**
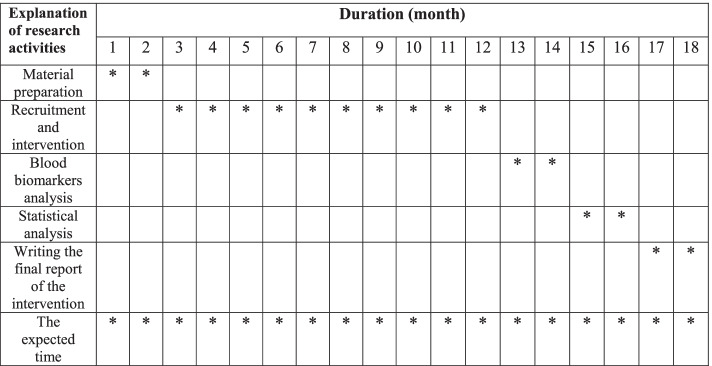
Timeline of the study (18 months have been expected for this trial)

This study will be an eight-week, multi-factorial, double-blind, randomized controlled trial with four groups. The study population will consist of overweight and obese women over 40 years old before menopause who will be selected from the staff of Shiraz University of Medical Sciences referring to the cohort center of the University for follow-up. In order to encourage people to participate in the study, announcements will be prepared in their workplaces and an expert will be present in the cohort center to inform them about the aims and health benefits of the study. The participants will be recruited if they have insufficient vitamin D intake. The study will be conducted on eligible individuals using synbiotic (500 mg, once a day) and vitamin D_3_ (50,000 IU, once a week) supplementation for 8 weeks. The outcome measurements will be done at the beginning and at the end of the eighth week (Figs. 1 and 2). The protocol has been written in line with the Standard Protocol Items: Recommendations for Interventional Trials (SPIRIT) checklist.

### Objectives and hypotheses of the study

The primary objective is to determine the effect of co-supplementation with synbiotic and vitamin D_3_ on the intestinal microbiome. As the second objective, the study will evaluate the relationship between alterations in microbiome and muscle strength, muscle function, and body composition in middle-aged overweight and obese women. In addition, in order to control confounders, dietary intakes, physical activity, and exposure to the sun will be assessed at baseline and at the end of the study.

### Participants

The study will be performed on 88 overweight and obese women (body mass index (BMI) ≥ 25 kg/m^2^) aged 40–55 years, having insufficient vitamin D intake, not having taken vitamin D supplements orally or by injection during the past 2–3 months, not having reached menopause, not having taken synbiotic, probiotic, and prebiotic supplements during the past 2–3 months, not having the indications of chronic diseases such as diabetes, cardiovascular disease (CVD), uncontrolled blood pressure, and liver and kidney disorders, not taking supplements containing vitamins, minerals, and omega-3 or fish oil, not having taken antibiotics during the past 3 weeks, not following a special diet, not having used the medications that affect weight over the past 2 months, and not having used steroidal and non-steroidal anti-inflammatory drugs, anticonvulsants, anti-cholesterol drugs, antacids, diuretics, and laxatives over the past 2 months. The exclusion criteria will consist of being influenced by any acute diseases during the study, being less than 90% compliant with the intervention, and not being willing to continue the study.

### Ethics and trial registration

The study protocol has been registered in the Iranian Registry of Clinical Trials (IRCT20090822002365N25) and has been conformed to the Declaration of Helsinki Guidelines. It has also been approved by the Ethics Committee of Iran University of Medical Sciences, Tehran, Iran (IR.IUMS.REC.1399.1177). Written informed consent forms will be signed by all the participants. At the end of the study, vitamin D supplementation will be provided for the individuals receiving vitamin D placebo during the trial. Additionally, all the participants will have the opportunity to receive diet and food recommendations from the nutritionist.

### Sample size

Due to the fact that the primary outcome of the research is gut microbiome assessment, the sample size was determined according to the study carried out by Most et al. [24], which evaluated gut microbiota composition in relation to the metabolic response to polyphenol supplementation in overweight individuals. Using the Standard Deviation (SD) of the logarithm of Firmicutes from that study (0.32), considering a difference equal to 1.25 SD (0.32 units of change in the logarithm of Firmicutes) between the two groups with the lowest and highest values of Firmicutes, and considering *α* = 0.05, *β* = 0.10, and a 10% dropout, a 22-subject sample size was estimated for each group. In total, 88 overweight and obese middle-aged women will be enrolled.

### Randomization

#### Sequence generation

To perform random allocation, permuted block randomization will be used by quadrilateral blocks. Considering the 88-subject sample size, 22 blocks will be generated using a website (www.sealedenvelope.com).

#### Allocation concealment mechanism

In order to blind all the investigators and participants, an individual not involved in the trial will make a randomized list assignment for the participants in each group and will label all the identical containers (in appearance and color) based on their numbers. Therefore, the participants will be unaware of the type of the intervention they receive. The random sequence will be unpredictable, as well.

The 88 participants will be allocated into four groups at a 1:1:1:1 ratio, as follows:

1. Receiving a vitamin D placebo soft gel containing paraffin (weekly) along with one capsule of synbiotic placebo containing 500 mg maltodextrin (daily) for 8 weeks
2. Receiving a 50,000 IU vitamin D soft gel (weekly) along with one capsule of synbiotic placebo containing 500 mg maltodextrin (daily) for 8 weeks
3. Receiving a vitamin D placebo soft gel containing paraffin (weekly) along with a 500 mg synbiotic capsule (daily) for 8 weeks
4. Receiving a 50,000 IU vitamin D soft gel (weekly) along with a 500 mg synbiotic capsule (daily) for 8 weeks

The synbiotic capsules (GeriLact) will contain seven bacteria species (*Bifidobacterium longum* 3.5 × 10^9^ colony-forming units [CFU], *Lactobacillus casei* 3.5 × 10^9^ CFU, *Lactobacillus acidophilus* 10^9^ CFU, *Bifidobacterium breve* 10^10^ CFU, *Lactobacillus rhamnosus* 7.5 × 10^8^ CFU, *Lactobacillus bulgaricus* 10^8^ CFU, and *Streptococcus thermophilus* 10^8^ CFU per 500 mg), and 38.5 mg fructooligosaccharide. They will be manufactured by ZistTakhmir Co., Tehran, Iran. In addition, the placebo capsules with 500 mg maltodextrin content will be provided by the same appearance and color as the synbiotic supplement. Vitamin D soft gels and placebos containing paraffin will be produced by Zahravi, Iran. All the supplements will be placed in identical containers. The participants will be instructed to use the products. At the end of weeks 4 and 8, they will be asked to return the empty containers in order to count the number of remaining supplements for compliance assessment. The participants will also be contacted during the study to check their tolerance and to record the possible side effects of the supplements.

#### Implementation

The potential participants will be invited to the study by SJ and SJM. Sequencing will be generated by M who is not going to assign the participants into the study.

#### Outcome measures

The primary outcome of the study will be the assessment of the intestinal microbiome and the secondary outcomes will include muscle strength, muscle function, and body composition.

At the beginning of the study, the demographic information will be gathered during an interview. In order to assess the dietary intake and compliance through the study, a three-day dietary recall questionnaire (including one weekend day and two weekdays) will be completed by a trained nutritionist at baseline and at the end of the intervention. Nutrient analysis will also be performed by Nutritionist IV software (First Databank, San Bruno, CA) modified for Iranian foods. Moreover, physical activity levels will be assessed by the short form of the International Physical Activity Questionnaire (IPAQ) at the beginning and end of the study. To evaluate sun exposure quantitatively, use will be made of the questionnaire in the research performed by Nikooyeh et al. [25]. It should be noted that the participants will be asked to maintain their usual diets and physical activities during the intervention.

Anthropometric measurements will be determined at baseline and eight weeks after the intervention. Body weight will be measured by minimal clothing and without shoes using a Seca scale with the accuracy of 0.1 kg. Height assessment will be done by a wall stadiometer with the precision of 0.1 cm without shoes. Then, BMI will be calculated as weight in kilograms divided by height in meters squared. Additionally, waist circumference will be measured in the narrowest area above the iliac crest using a flexible tape meter with the accuracy of 0.1 cm.

Body composition including total body, arm and leg lean tissue mass, fat-free mass (FFM), total body fat mass (FM), bone mineral content (BMC), visceral fat area (VFA), and protein content will be determined using a segmental multi-frequency Bioelectrical Impedance Analysis (BIA) InBody S10 analyzer (BioSpace Co., Ltd., South Korea) at the beginning of the study and after the intervention. To obtain the appendicular skeletal muscle mass (ASM), the sum of arms’ and legs’ muscle mass will be calculated. Besides, skeletal muscle mass index (SMI), as an indicator of sarcopenia, will be computed via dividing ASM by height in meters squared.

Upper and lower muscle strengths will be assessed based on handgrip and knee extension strengths using a dynamometer at the beginning and end of the study. Handgrip strength (HGS) will be evaluated for each participant by squeezing a hydraulic hand dynamometer (model MSD, Sihan, Korea) three times in seated position with 1-min intervals. The highest attempt of the dominant hand will be considered for the subsequent analyses. Knee extension strength will be measured in the dominant foot using a portable handheld dynamometer (Digital Force Gauge: SF-500, China) three times with 1-min intervals to rest in the seated position. The three values will be recorded, and the maximum will be defined as the knee extension strength. All the values will be expressed in kilograms.

Physical performance will be characterized using Timed Get-Up-and-Go (TGUG) test. The test will be started at the word “go” and the participants will be required to stand up, walk over a 3-m distance, turn around, walk back, and sit down. The time will be recorded by a chronometer and TGUG will be reported in meters per second. To prevent the measuring error, all strength and performance variables will be determined by one person. All the measurements will be made at the beginning and end of the study.

At baseline and end of the eighth week, stool samples will be collected and transferred to the laboratory using ice packs. The samples will be stored at – 80 °C until analysis. Favorgen Biotech stool mini kit (Favorgen Biotech Corp., Taiwan) will be used for fecal DNA isolation. In addition, real-time polymerase chain reaction (PCR) will be done for quantifying Bifidobacterium, Enterococcus, Firmicutes, Bacteroidetes, and Lactobacillus. The required primers (forward and reverse primers) and probe sequences [26] for the five real-time PCR assays have been provided in Table 1.

**Table 1:** Primers and probes for detection of Bifidobacterium, Enterococcus, Firmicutes Bacteroidetes, and Lactobacillus

Targets	Primer and probe sequences
Bifidobacterium	FP: GCGTGCTTAACACATGCAAGTC RP: CACCCGTTTCCAGGAGCTATT Probe: TCACGCATTACTCACCCGTTCGCC
Enterococcus	FP: AGAAATTCCAAACGAACTTG RP: CAGTGCTCTACCTCCATCATT Probe: TGGTTCTCTCCGAAATAGCTTTAGGGCTA
Firmicutes	FP: GTCAGCTCGTGTCGTGA RP: CCATTGTAKYACGTGTGT Probe: GTCAANTCATCATGCC
Bacteroidetes	FP: AGCAGCCGCGGTAAT RP: CTAHGCATTTCACCGCTA Probe: GGGTTTAAAGGG
Lactobacillus	FP: TACATYCCAACHCCAGAACG RP: AAGCAACAGTACCACGACCA Probe: AAGCCATTCTTRATGCCAGTTGAA

*FP* forward primer, *RP* reverse primer

#### Statistical analysis

Statistical analysis of the data will be performed using the SPSS 22.0 software (SPSS Inc., Chicago, IL). Initially, the Shapiro–Wilk test will be used for the evaluation of normality. The data will be reported as percentages for qualitative variables and mean ± SD for quantitative variables. Categorical variables will be compared using chi-square test. In addition, the baseline mean differences will be assessed using the analysis of variance (ANOVA). For assessing pairwise differences between the study groups, Tukey’s post-hoc test will be performed. Analysis of covariance (ANCOVA) will also be used to evaluate the intervention effects between the study groups with adjustment for baseline values. A logarithmic correction will be done for skewed data before analysis. Moreover, paired-sample *t*-test and Wilcoxon test will be used to compare the mean values of normally distributed and skewed data within groups after the intervention. To analyze the microbiome data, the fold expression change will be calculated. It should be noted that at 10% or higher dropouts, the intention to treat (ITT) analysis will be conducted. *P* < 0.05 will be considered statistically significant.

#### Protocol amendments

Any modification to the study protocol including changes in the study objectives, study design, patient population, sample size, study procedures, or significant administrative aspects that may affect the conduct of the study, patients’ potential benefits, or patient safety will require a formal amendment to the protocol. Such amendments will be agreed upon by the Department of Nutrition, School of Public Health, Iran University of Medical Sciences, and will be approved by the Ethics Committee of Iran University of Medical Sciences prior to implementation. Administrative changes of the protocol are minor corrections and/or clarifications that have no effects on the way the study is to be conducted. These administrative changes will be agreed upon by Iran University of Medical Sciences. The Ethics Committee of Iran University of Medical Sciences may be notified of the administrative changes at the discretion of the Department of Nutrition, School of Public Health, Iran University of Medical Sciences.

## Discussion

The exact time of onset and the extent of muscle loss are not clear. However, evidence has suggested that muscle wasting begins progressively around the age of 40 years and obesity can exacerbate muscle function and mobility. Additionally, studies have demonstrated that older and obese people are prone to intestinal dysbiosis, which is associated with the impairment of intestinal microbiome homeostasis, increased intestinal permeability, increased circulating endotoxin levels, and inflammation. Furthermore, dysbiosis reduces the expression of phosphorylated adenosine monophosphate-activated protein kinase (AMPK) in skeletal muscle and liver, leading to weight gain [27]. The contribution of AMPK activity reduction to aging and reactive oxygen species (ROS) production has been advanced, as well [28].

Various studies have indicated that probiotics are involved in stimulating FOXN1 gene expression and reducing inflammation. Probiotics also reduce the level of atherogen-1 (a marker of muscle atrophy), increase access to amino acids, and modulate the production of inflammatory cytokines. They are also involved in insulin sensitivity, stimulation of beta-pancreatic cells, and improvement of mitochondrial function by producing SCFA and reducing ROS. Moreover, adiposity in the liver and skeletal muscles is regulated by gut microorganisms via AMPK levels [29]. On the other hand, vitamin D improves the intestinal microbiome function and homeostasis. Vitamin D plays a strong anti-inflammatory role by reducing Th1, Th17, and inflammatory cytokines in the gastrointestinal tract. Recent studies have proved that vitamin D signaling via VDR plays an important role in regulating myoblast proliferation and differentiation. Moreover, the role of vitamin D in facilitating muscle anabolism and maintaining muscle mass has been considered recently.

Altogether, the current study aims to increment evidence regarding the aforementioned issues. Hence, it will assess the impact of the co-supplementation of synbiotic and vitamin D on the intestinal microbiome, muscle strength, muscle function, and body composition in middle-aged overweight and obese women.

### Trial status

This study was started on 20 July 2021 and is at the preparation stage.

## Data Availability

Not applicable.

## Abbreviations

SCFA: Short-chain fatty acids
NF-κB: Nuclear factor kappa B
TLR: Toll-like receptor
BMI: Body mass index
CVD: Cardiovascular disease
IRCT: Iranian Registry of Clinical Trials
IPAQ: International physical activity questionnaire
FFM: Fat-free mass
FM: Fat mass
BMC: Bone mineral content
VFA: Visceral fat area
BIA: Bioelectrical impedance analysis
ASM: Appendicular skeletal muscle mass
SMI: Skeletal muscle mass index
HGS: Handgrip strength
TGUG: Timed get-up-and-go
AMPK: Phosphorylated adenosine monophosphate-activated protein kinase
ROS: Reactive oxygen species
VDR: Vitamin D receptor
